# Removal of redundant contigs from *de novo* RNA-Seq assemblies via homology search improves accurate detection of differentially expressed genes

**DOI:** 10.1186/s12864-015-2247-0

**Published:** 2015-12-04

**Authors:** Hanako Ono, Kazuo Ishii, Toshinori Kozaki, Isao Ogiwara, Motoki Kanekatsu, Tetsuya Yamada

**Affiliations:** United Graduate School of Agricultural Science, Tokyo University of Agriculture and Technology, Fuchu, Tokyo 183-8509 Japan; Department of Applied Biological Science, Faculty of Agriculture, Tokyo University of Agriculture and Technology, Fuchu, Tokyo 183-8509 Japan

**Keywords:** Plant breeding, Unsequenced plant genomes, *de novo* assembly, RNA-Seq, Redundant contigs, Genome editing, Genetic modification, Transcriptome, Trinity

## Abstract

**Background:**

For plant species with unsequenced genomes, cDNA contigs created by *de novo* assembly of RNA-Seq reads are used as reference sequences for comparative analysis of RNA-Seq datasets and the detection of differentially expressed genes (DEGs). Redundancies in such contigs are evident in previous RNA-Seq studies, and such redundancies can lead to difficulties in subsequent analysis. Nevertheless, the effects of removing redundancy from contig assemblies on comparative RNA-Seq analysis have not been evaluated.

**Results:**

Here we describe a method for removing redundancy from raw contigs that were primarily created by *de novo* assembly of *Arabidopsis thaliana* RNA-Seq reads. Specifically, the contigs with the highest bit scores were selected from raw contigs by a homology search against the gene dataset in the TAIR10 database. The two existing methods for removal of redundancy based on contig length or clustering analysis used to eliminate redundancies from raw contigs. Contig number was reduced most effectively with the method based on homology search. In a comparative analysis of RNA-Seq datasets, DEGs detected in contigs that underwent redundancy removal via the homology search method showed the highest identity to the DEGs detected when the TAIR10 gene dataset was used as an exact reference. Redundancy in raw contigs could also be removed by a homology search against integrated protein datasets from several plant species other than *A. thaliana*. DEGs detected using contigs that underwent such redundancy-removed also showed high homology to DEGs detected using the TAIR10 gene dataset.

**Conclusion:**

Here we describe a method for removing redundant contigs within raw contigs; this method involves a homology search against a gene or protein database. In principal, this method can be used with unsequenced plant genomes that lack a well-developed gene database. Redundant contigs were not removed adequately via either of two existing methods, but our method allowed for removal of all redundant contigs. To our knowledge, this is the first reported improvement in accurate detection of DEGs via comparative RNA-Seq analysis that involved preparation of a non-redundant reference sequence. This method could be used to rapidly and cost-effectively detect useful genes in unsequenced plants.

## Background

Genome editing technology allows modification of target genes and introduction of foreign genes into a specific genomic region [1, 2]. To accelerate plant breeding using such technology, it is necessary to identify useful genes that can improve target traits. For example, the objective of breeding golden rice, which is high in the vitamin A precursor beta-carotene, was achieved by identifying *CrtI* and *Psy* as useful genes from vitamin A synthesis-related genes via comprehensive analysis [3]. Thus, comprehensively detecting all genes involved in a target trait is considered an important first step in identifying genes important to a breeding objective.

Analysis of differentially expressed genes (DEGs) by microarrays [4–6] or next generation sequencing [7–9] has been used to comprehensively detect all genes involved in several different traits. However, analysis of genes with low transcript abundance via microarray technology is difficult because the microarray detection limit is relatively high [10]. In addition, microarrays can analyze only the particular set of genes arrayed on a DNA chip, which must therefore contain the gene of interest. In contrast, transcriptome analysis using next generation sequencing (i.e. RNA-Seq) can detect all expressed genes without relation to their transcript abundance [10]. Thus, RNA-Seq is a more suitable method for comprehensive DEG detection that is aimed at identification of useful genes, but RNA-Seq requires the whole genome of the target species as a reference sequence.

The reference sequence for RNA-Seq can be obtained easily if the genome of the target species has been sequenced [10], but must be prepared another way if the genome is unsequenced. Sequencing of the whole genome in the target species is one solution, especially as the cost of genome sequencing becomes lower [11]. However, genome sequencing of wild species in which the existence of useful genes is unclear has a higher cost-to-benefit ratio than does sequencing of cultivated species. Also, genome sequencing is extremely difficult in allopolyploid species [12]. For these reasons, construction of a reference sequence by *de novo* assembly of RNA-Seq reads has been tried repeatedly [13–15].

Several programs for *de novo* assembly of RNA-Seq reads (e.g., Velvet, Trinity, and SOAP *de novo*) have been developed [16–19]. Trinity is designed to assemble short reads [20], and is expected to be suitable for construction of reference sequence from RNA-Seq reads. Indeed, cDNA contigs assembled from RNA-Seq reads using such assemblers have been used as reference sequence for comprehensive gene detection via RNA-Seq [13–15]. Previously, methods for improvement of *de novo* assembly were thoroughly investigated [21]; however, the number of cDNA contigs was still significantly higher than the number of estimated genes. This suggests that multiple contigs are formed for individual genes because of assembly of incomplete reads; these duplicate contigs represent redundancy in the contig assembly. The existence of such contig redundancy is likely to pose difficulties in comparative analysis aimed at detecting DEGs [22]. If redundant contigs are used as a reference sequence for RNA-Seq data, several contigs derived from the same gene would be incorrectly identified as different DEGs.

Several approaches for removal of redundant contigs have been proposed. When RNA-Seq reads are assembled with Trinity, a group of integrated contigs (called a subcomponent) is formed when considering splicing variants. Yang et al. tried to remove redundancy by selecting the longest contig from each subcomponent formed by Trinity [15]. Several groups used CD-HIT to remove redundant contigs from *de novo* contig assemblies by removing contigs that showed homology; CD-HIT is a program that selects as a representative sequence the longest contig in each cluster of contigs [21, 23–25]. Though both approaches led to fewer, longer contigs, the number of contigs was still large; moreover, the studies did not assess whether removal of redundant contigs via these approaches actually improved the accuracy of DEG detection via comparative RNA-Seq analysis. Therefore, developing an effective method for removing redundant contigs should specifically focus on improving accurate detection of DEGs.

To re-create an accurate reference sequence for detecting useful genes, several issues should be considered. The set of contigs should contain no redundancy; in other words, only unique contigs should remain, even if removal of redundant contigs results in an incomplete set of contigs. To create a redundancy-free reference sequence, we used BLAST, the Basic Local Alignment Search Tool [26], which has been used for removing redundancy together with the alignment program, CAP3, as well as for annotation [27]. In these BLAST searches, it is assumed that there will be contigs exhibiting homology with other contigs, and such contigs may be regarded as redundant. Complete removal of all redundant contigs by this method is expected to greatly improve accurate detection of DEGs.

In this study, we evaluated the efficacy of removing redundant contigs from a raw contig assembly of *A. thaliana* RNA-seq reads using the well-developed *A. thaliana* gene database. We also compared two existing methods for removal of redundant contigs with our homology search-based method using BLAST alone with regard to accurate detection of DEGs in a comparative RNA-Seq analysis; one of these existing methods selects the longest contig from each subcomponent; the other method used CD-HIT to remove all shorter contigs that have homology with a longer contig. Our method selected contigs with the highest bit score in a BLAST search. Additionally, for application of our method to plant species with unsequenced genomes, we removed redundant contigs from the set of raw contigs via BLAST searches with protein datasets of various plants instead of *A. thaliana* gene datasets and confirmed the effect on accuracy of DEGs detection.

## Methods

### Data

Datasets of *A. thaliana* RNA-Seq reads for *de novo***c**ontig assembly and comparative analysis were downloaded via the SRA download page (http://www.ncbi.nlm.nih.gov/sra). Transcript sequence datasets from *A. thaliana* (TAIR10; [28]) were downloaded via the TAIR database (http://www.arabidopsis.org) for detection and removal of redundant contigs. All protein-coding transcripts were selected from the database, and a set of 35,385 transcript sequences is referred to here as the gene dataset. GO terms for *A. thaliana* were downloaded via the AgriGO download page (http://bioinfo.cau.edu.cn/agriGO/download.php) [29]. Protein datasets of *Carica papaya*, *Cannabis sativa*, *Glycine max*, *Medicago truncatula*, *Oryza sativa*, *Prunus persica*, *Populus trichocarpa*, *Ricinus communis*, *Sorghum bicolor*, *Setaria italica*, *Solanum lycopersicum*, *Selaginella smoellendorffii*, *Vitis vinifera*, and *Zea mays* were also downloaded via the AgriGO download page. Protein datasets were used to remove redundant contigs. The protein datasets for each of the other 14 species (listed above) were combined; the combined dataset was named Plant DB. A non-duplicative database based on Plant DB was formed using the CD-HIT program [23] with an identity setting of 0.5; this database was designated the PlantClust50 DB.

### *De novo* assembly of RNA-Seq reads

All reads for the RNA-Seq datasets derived from roots, floral buds, or seedlings of 10-day-old *A. thaliana* seedlings (Table 1) were used for de novo assembly using the Trinity platform [18]. Contigs assembled with a minimum-contig-length parameter setting of 120 nucleotides; the default settings were used for all other parameters. Contigs generated via this primary assembly process were considered raw contigs.

**Table 1:** Summary of reads used for de novo assembly

Library	Source	Total number of reads	Number of bases (giga base)	Average length (bases)
SRR314813	11-day-old seedling, Col-0	28,783,170	2.4	83.4
SRR314814^a^	10-day-old root, Col-0	31,362,126	2.6	82.9
SRR314815^a^	stage 12 floral bud, Col-0	28,988,204	2.4	82.8
SRR314816	11-day-old seedling, Can-0	27,886,057	2.3	82.5
SRR314817	10-day-old root, Can-0	33,556,983	2.8	83.4
SRR314818	stage 12 floral bud, Can-0	27,798,328	2.3	82.7
Total		178,374,868	14.8	83.0

^a^ also used for short-read mapping

Col-0 and Can-0 indicates Columbia and Canary Island, the ecotype of *A. thaliana* respectively

### Detection of redundancies in raw contigs

Homology searches of raw contigs were performed locally using the blastn algorithm and the TAIR10 gene dataset or using blastx and protein datasets, i.e. Plant DB or PlantClust50 DB; neither an e-value nor an identity cut-off was used in these searches. Any contig showing homology to any gene or protein was designated a hit contig. If the contig showed homology to a single gene or protein, it was classified as a unique hit contig. Any contig that did not show homology to any gene was classified as a no-hit contig. Contigs that showed homology to a single gene along with other contigs were categorized as multiple hit contigs. Based on the homology search results, the gene or protein corresponding to each contig was defined.

### Removal of redundancies from raw contigs

We used three approaches to remove redundant contigs from the set of raw contigs. 1) The longest contigs were selected from each subcomponent of raw contigs generated by Trinity as described in Yang et al. [15]. 2) The CD-HIT program was used to generate clusters of raw contigs; the identity setting was 0.9 and the default parameters used were described previously [24, 25]; the longest sequence in each cluster was identified and designated a clustered contig. 3) The contig with the highest BitScore for each respective gene or protein was selected from raw contigs based on the results of homology searches that were designed to detect redundant contigs; this highest-scoring contig was designated an annotated contig. Annotated contigs were also selected based on the results of blastx algorithm homology searches with raw contigs as query sequences and the protein datasets of Plant DB or PlantClust50 DB as the reference dataset, and based on the dataset, each redundancy-removed contig was designated a Plant DB contig or a PlantClust50 DB contig.

### Detection of DEGs by comparative analysis of RNA-Seq datasets

SRR314814 and SRR314815, which are two RNA-Seq datasets from *A. thaliana*, were mapped with the Bowtie2 aligner [30] to the TAIR10 gene dataset, a raw contig set, and a redundancy-removed contig set with the options “-q -phred33 --sensitive-local -N 1”. The number of reads per kilobase of exon per million mapped reads (RPKM) of each gene or contig was calculated according to Mortazavi et al. [31]. The RPKM value was added to 1 as a correlation value; the log2-fold change between datasets was then calculated. The log2-fold change for each contig was plotted against the log2-fold change of the corresponding gene in TAIR10. To determine fold-change in expression for each unique contig, correlation coefficients were calculated between the gene dataset and raw contigs or the gene dataset and each redundancy-removed contig. Genes and contigs with a fold-change greater than one were defined as DEGs or differentially expressed contigs (DECs), respectively.

### Evaluation of co-identity between DEGs and DECs

Co-identities between DEGs and DECs were evaluated using gene ID and Gene Ontology (GO) analysis. DECs were annotated with the corresponding *A. thaliana* gene ID based on the homology search results. Then, the gene IDs of DEGs and DECs were compared. The number and the ratio of DECs that had the same gene ID as DEGs were calculated. Analysis of GO slim term enrichment of DEGs and DECs was performed using the BLAST2go program [32]. The distribution of *A. thaliana* GO slim terms in the DEG set and was compared that in the DEC set to assess which methods were optimal for removing contig redundancy. The Kolmogorov-Smirnov test was used to quantify the distance between the DEGs and the DECs with regard to GO term distribution. In this analysis, the null hypothesis was that the two distributions were the same. If the *p*-value was under 0.05, the null hypothesis could be rejected. The difference between the DEGs and the DECs with regard to each GO slim annotation count was evaluated by Fisher’s exact test.

## Results

### Redundancies in raw contigs

Number, average length, and N50 values were compared between the gene dataset and raw contigs assembled *de novo* (Table 2). The number of raw contigs (62,339) was larger than the number of genes in the dataset (35, 385). Both the mean length and mean N50 value for the raw contig set were smaller than those of the gene dataset (Table 2). The number of contigs exhibiting homology with a gene (hit contigs) was 58,376 (93.64 % of total contigs) (Table 3). Of these contigs, 10,119 (16.23 %) were unique-hit contigs, and 48,257 (77.41 %) were multiple hit contigs.

**Table 2:** Summary of gene dataset and reference contigs derived from de novo assembly of RNA-Seq reads in *A. thaliana*

	Gene dataset	Raw Contigs	Longest Contigs	Clustered Contigs	Annotated Contigs	Plant DB Contigs	PlantClust50 DB Contigs
Number	35,385	62,339	58,007	59,405	23,873	42,467	26,766
Min. length (base)	22	121	121	121	121	121	121
Median length (base)	1383	285	275	281	599	317	351
N50 (base)	1814	739	699	711	1042	826	987
Mean length (base)	1535	475	457	464	757	520	589

**Table 3:** Summary of homology searches of contigs against TAIR10 gene dataset using BLAST

	Raw Contigs	Longest Contigs	Clustered Contigs	Annotated Contigs	Plant DB Contigs	PlantClust50 DB Contigs
Hit contigs	58,376 (93.64)*	54,175 (93.39)	55,857 (94.03)	23,873 (100)	40,179 (94.61)	24,784 (92.59)
Unique hit contigs	10,119 (16.23)	10,676 (18.40)	10,571 (17.79)	23,873 (100)	11,382 (26.80)	12,398 (46.32)
Multiple hit contigs	48,257 (77.41)	43,499 (74.99)	45,286 (76.23)	0 (0)	28,796 (67.81)	12,386 (46.28)
No hit	3963 (6.36)	3832 (6.61)	3548 (5.97)	0 (0)	2289 (0.05)	1982 (0.07)
Total	62,339 (100)	58,007 (100)	59,405 (100)	23,873 (100)	42,467 (100)	26,766 (100)

* Values in parentheses are percentages of all contigs

### Comparison of three methods for removal of redundant contigs from the set of raw contigs

Each of three methods was used to remove redundant contigs from the raw contig set, and each method produced a distinct set of contigs (longest contigs, clustered contigs, or annotated contigs). Comparisons of contig number, average length, and N50 value for these three contig sets are shown in Table 2. Mean length and N50 value were higher for the annotated contig set than for the longest contig or clustered contig set. Next, all contigs in each contig set were annotated through homology searches against the gene dataset (Table 3). Next all multiple-hit contigs were removed from the annotated contig set, so that all hit contigs in this set were unique-hit contigs. In contrast, when all multiple-hit contigs were removed from longest contig or clustered contig set, the number and ratio of multiple-hit contigs decreased, while the number of unique-hit contigs slightly increased (Table 3).

### Comparison of redundancy-elimination methods with regard to DEC detection

**Fig. 1:**
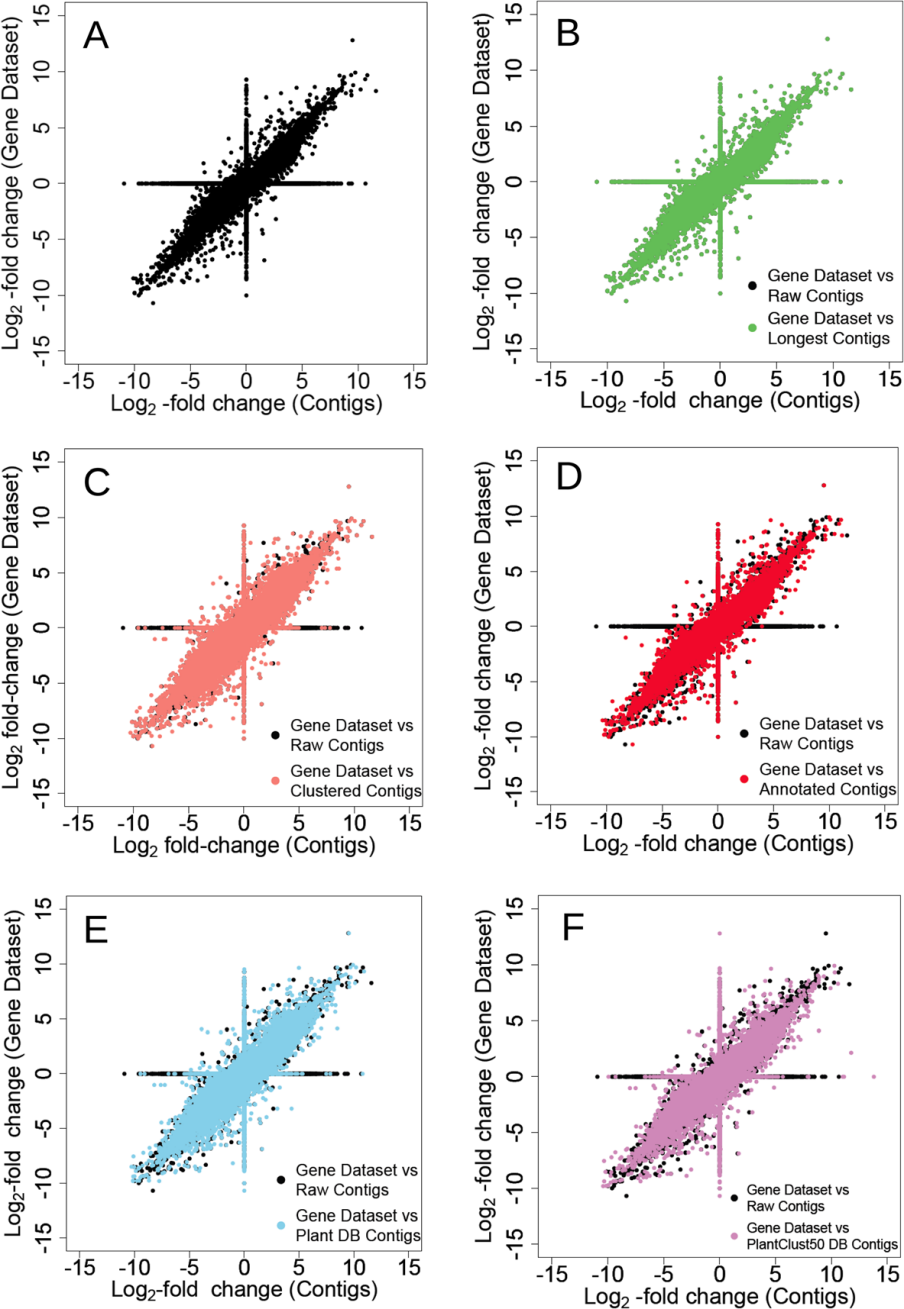
Scatter plots of log2-fold changes in gene dataset vs. various reference sequences. Values for the log2-fold changes in genes were calculated accurately, but erroneous contigs were included. **a** Scatter plot of log2-fold changes in the gene dataset vs. primary assembled contigs (raw contigs). Scatter plot of log2-fold changes in gene dataset vs. various contig groups after removing redundant contigs by (**b**) Longest method, (**c**) Clustered method, (**d**) Annotation using the gene dataset, (**e**) Annotation using combined plant protein database (Plant DB), (**f**) and non-duplicated Plant DB (PlantClust50 DB). Panels **b–f** overlay data points of panel **a**, indicated by *black dots*

The gene dataset and individual contig groups were each used as reference sequence for comparative analysis of RNA-Seq datasets (SRR314814 and SRR314815 in Table 1). A scatter plot of log2-fold changes was created (Fig. 1), and the correlation coefficient between the gene dataset and each contig group was calculated (Table 4). The correlation coefficient between the gene dataset and raw contigs was 0.60 (Table 4). The highest correlation coefficient was found between the gene dataset and the annotated contig set (Fig. 1d, Table 4). In the scatter plot of log2-fold changes in raw contigs vs. the gene dataset, there were data points falling along X = 0 and Y = 0 (Fig. 1a). The data points falling along X = 0 indicated that no contig correlated with any expressed gene. The data points falling along Y = 0 indicated that these contig exhibited no homology with genes. In the scatter plot of annotated contigs, the number of data points with Y = 0 was very small (Fig. 1d); this result indicated that contigs that did not correspond to any genes had been removed from the annotated contig set. The longest contig set had the most data points where Y = 0 (Fig. 1b). For the clustered contig set, the number of data points with Y = 0 was lower than for the raw contigs; nevertheless, there were still many Y = 0 data points (Fig. 1c).

**Table 4:** Summary of co-identity in redundancy-removed contigs

	Raw Contigs	Longest Contigs	Clustered Contigs	Annotated Contigs	Plant DB Contigs	PlantClust50 DB Contigs
Correlation coefficient of log2-fold change between gene dataset and reference sequences	0.60	0.60	0.88	0.88	0.83	0.73
No. of DECs exhibiting co-identity with DEGs^a^	8602 (73.8 %)	8331 (74.2 %)	8506 (75.8 %)	8303 (74.0 %)	7594 (67.7 %)	5878 (52.4 %)
No. of DECs redundant or not exhibiting co-identity with DEGs^b^	15,706 (64.6 %)	14,219 (63.1 %)	28,640 (77.1 %)	1372 (14.2 %)	9052 (54.4 %)	4809(45.0 %)
P-value^c^	0.44	0.20	0.01	1.00	0.93	0.93
Fisher’s exact test^d^	30	30	31	4	29	17

^a^ Number of contigs identical to DEGs in gene dataset (values in parentheses are percentage of all DEGs identical to DECs)

^b^ Number of contigs not corresponding DEGs in gene dataset (values in parentheses are percentage of contigs without corresponding genes in contig group)

^c^ Correlation between gene database and contig group in GO slim term distribution calculated by Kolmogorov–Smirnov test

^d^ No. of GO terms significantly different from gene dataset in annotation count

### Evaluation of accuracy of DEC detection

**Fig. 2:**
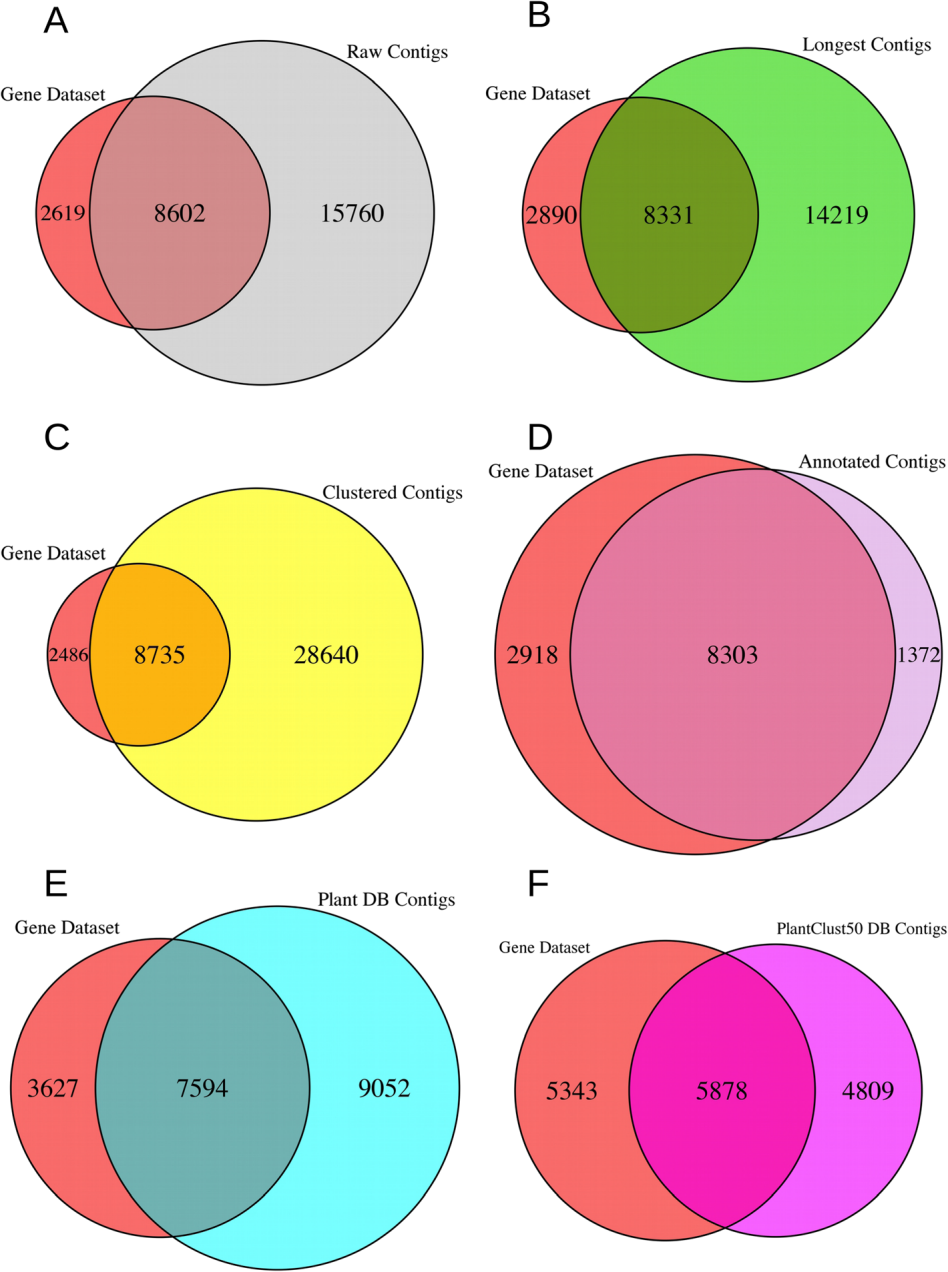
Accuracy of detection of differentially expressed genes (DEGs) in reference sequences. Shown are the number of DEGs or differentially expressed contigs (DECs). The gene dataset indicates DEGs in all panels. **a** Raw contigs: DECs in raw contigs, (**b**) Longest contigs: DECs in longest contigs, (**c**) Clustered contigs: DECs in clustered contigs, (**d**) Annotated contigs: DECs in annotated contigs, (**e**) Plant DB contigs: DECs in Plant DB contigs, (**f**) PlantClust50 contigs: DECs detected in PlantClust50

Gene ID was used to evaluate co-identity between DEGs and DECs for each contig set (Table 4, Fig. 2). Of the 24,362 DECs in the raw contig set, 8602 DECs were identical to at least one DEG, and these 8602 DECs accounted for 76.7 % of all DEGs (Fig. 2a). For each other contig set, the number of DECs identical to a DEG was almost the same as the number for the raw contig set, i.e., 8331, 8506, and 8303 for the longest contig, the clustered contig, and annotated contig sets, respectively (Fig. 2b–d). Conversely, the number of DECs not identical with a DEG differed greatly between contig groups, i.e., 14,219, 28,640, and 1372 DEC were not identical with any DEG for the longest contig, clustered contig, and the annotated contig sets, respectively (Fig. 2b–d).

**Fig. 3:**
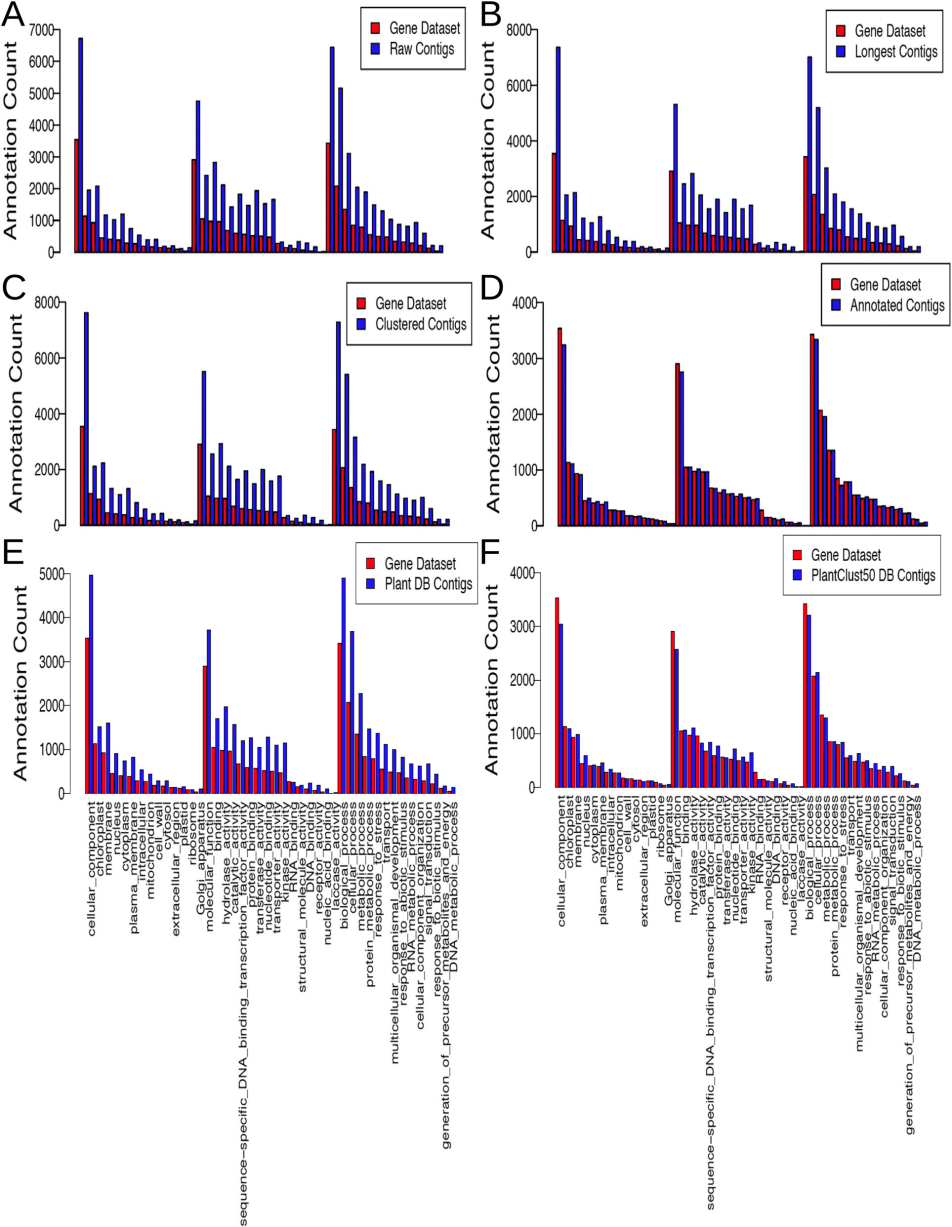
Comparison of *A. thaliana* GO slim term distribution of differentially expressed genes and differentially expressed contig. The number of DEGs or DECs assigned to the GO terms is plotted. Red bars and blue bars indicate DEGs and DECs, respectively. The reference sequences were, from top to bottom, (**a**) Raw contigs, (**b**) Longest contigs, (**c**) Clustered contigs, (**d**) Annotated contigs*,* (**e**) Plant DB contigs, and (**f**) PlantClust50 contigs

**Fig. 4:**
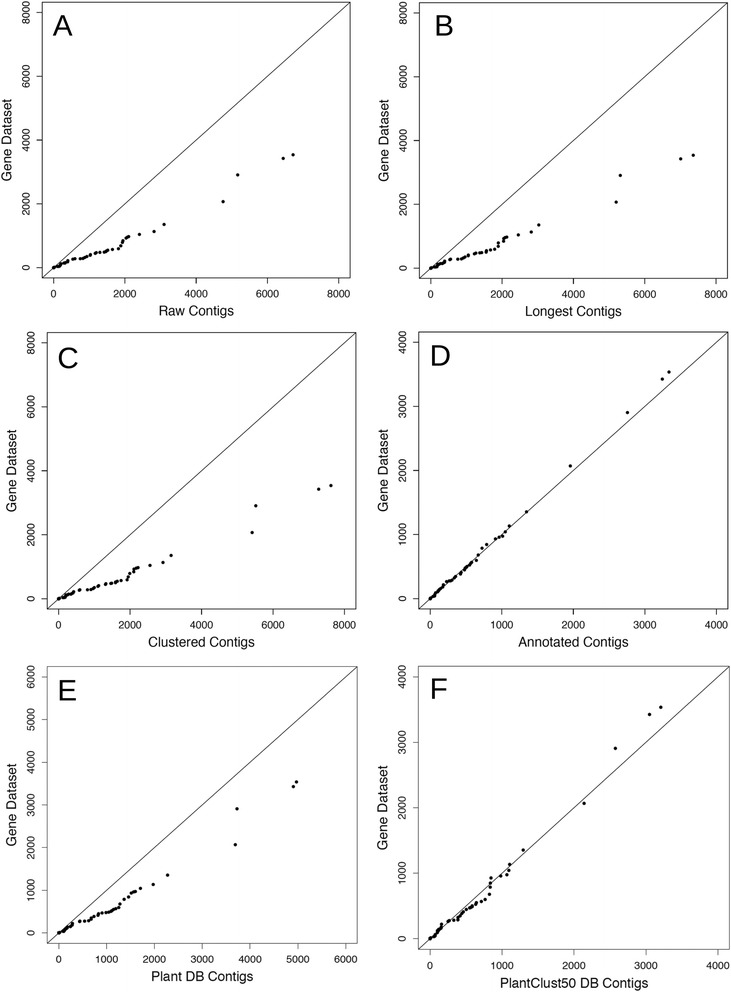
Conformity of annotation count of *A. thaliana* GO slim terms in differentially expressed contigs to differentially expressed genes. Quantile-quantile-plot of gene dataset vs. DECs detected through reference sequences: (**a**) Raw contigs, (**b**) Longest contigs, (**c**) Clustered contigs, (**d**) Annotated contigs, (**e**) Plant DB contigs, (**f**) PlantClust50 contigs. X-axis and y-axis indicate the number of DEGs and DECs in each GO term, respectively

Co-identity was also evaluated from comparison of the *A. thaliana* GO slim term distribution of DECs and DEGs (Figs. 3 and 4). A bar graph shows that, for the raw contig set, the annotation count for the GO slim terms in the DECs detected was larger than the annotation count of GO slim terms in DEGs (Fig. 3a). The distribution of GO slim terms in DEGs closely fitted that in DECs detected by annotated contigs (Fig. 3d). A quantile-quantile plot showed that the GO slim term distribution of DEGs best fit that of DECs detected by annotated contigs (Fig. 4d). Fisher’s exact test showed that the number of GO slim terms that were significantly different in annotation count between DECs and DEGs was least for the annotated contig set (Table 4).

### Removal of redundancies for unsequenced plants

Redundant contigs were also removed from the raw contig set via homology searches with duplicative or non-duplicative protein datasets of various plants instead of the *A. thaliana* gene dataset. Comparisons (the number, the correlation coefficient for fold change, co-identity, and p-value in the GO distribution of DECs) between the two resulting contig sets (Plant DB contigs and PlantClust50 DB contigs) are shown in Tables 2 and 4. The number of PlantClust50 DB contigs was 26,766, which was closer to the number of transcripts in the gene dataset (35,385) than the number of Plant DB contigs (42,463). A non-duplicative database based on Plant DB was also formed with an identity setting of 0.8 and 0.3. 35,385 contigs were generated with an identity setting of 0.8 (data not shown). The analysis took an enormous amount of time when the identity was set to 0.3. Thus the analysis was interrupted.

The scatter plot of the relationship of log2-fold change in the gene dataset and contig groups showed that the number of data points with Y = 0, which indicated contigs that lacked any corresponding genes, decreased in both Plant DB contigs and PlantClust50 DB contigs (Fig. 1e, f). The log2-fold changes in the gene dataset and in Plant DB contigs or PlantClust50 DB contigs were highly correlated, and similar to those of the annotated contig set (Table 4). Conversely, the number of DECs identical to DEGs in the Plant DB contigs and PlantClust50 DB contigs was lower than those in the raw contig and annotated contig sets (Table 4, Fig. 2e, f). The GO distribution in DECs detected by PlantClust50 DB contigs more closely fit the distribution in DEGs than the distribution in DECs detected by Plant DB contigs (Table 4, Figs. 3e, f and 4e, f). Fisher’s exact test revealed that the contig set that differed the least from the gene dataset in annotation count of GO slim terms was the PlantClust50 DB contig set (Table 4). The 0.8 setting in forming non-duplicative database did not increase the number of DECs assigned to the GO terms in the contigs; 28 GO slim terms were significantly different in annotateion count between DECs and DEGs in PlantClust DB.

## Discussion

### Creation of redundant contigs by incomplete assembly

The number of contigs constructed by *de novo* assembly (raw contigs) was larger than the total number of genes in the *A. thaliana* gene dataset (Table 2). However, the number of contigs exhibiting homology with genes was smaller than the total number of genes in *A. thaliana*. The main cause of this discrepancy was due to multiple-hit contigs, which were sets of contigs exhibiting homology to one gene, and thus regarded as redundant. Such contigs accounted for 77 % of all contigs (Table 3). The average length and N50 values of raw contigs were smaller than those of the gene dataset (Table 2). Thus, the redundancy detected in the raw contig set was presumably caused by incomplete contigs reconstructed from insufficient reads. This assumption was supported by the observation that when the RPKM of a gene was under 30, short and incomplete sequences were likely to be created (data not shown). Thus, it was predicted that contigs would be constructed more accurately with an increase in the read number. However, even if the RPKM of a gene was over 30, multiple-hit contigs emerged. This was presumably caused during next generation sequencing by limitations to *de novo* assembly. For example, AT-rich sequences were difficult to read [33], and repeat sequences were difficult to assemble [34]. Thus, a solution for removing redundant contigs, rather than simply increasing read number, was required. On the other hand, the contigs constructed by *de novo* assembly included some not exhibiting any homology with known genes. These were considered contigs with sequencing errors created due to failure in assembling reads. Such erroneous contigs should also be removed.

### Strategies for removal of redundant contigs

We discovered that redundant contigs resulted from multiple contigs constructed from reads from single genes. Hence, selecting one contig for each gene was needed to remove redundant contigs. Until recently, methods to remove redundant contigs have been studied, for example picking the longest contig of each subcomponent [15] and of each cluster [24, 25]. However, the effect of removing redundant contigs by such methods on RNA-Seq analysis had not been expressly evaluated. In this study, a homology search-based method using BLAST alone was developed and tested, and the effects of each distinct method on removing redundancy was evaluated. In strategies using clustered contigs or longest contigs, contig numbers were not reduced, and average contig length and N50 values did not increase compared with the raw contig set (Table 2). The number of multiple-hit contigs also did not decrease (Table 3). These results showed that these two methods were not suitable for removing redundancy. In contrast, using annotated contigs, the contig number decreased greatly, and the mean contig length and N50 value each increased (Table 2). Additionally, multiple-hit contigs and no-hit contigs disappeared (Table 3). Thus, these results suggested that our proposed method was optimal for removing redundant contigs and erroneous contigs. The clustered contig set was created by selecting the longest contig in an individual cluster consisting of contigs having 80 % or more identity. However, when multiple and partial clusters were created from the reads derived from one gene, no homology was exhibited among these clusters. Thus, the contigs in such clusters were not selected, and remained as redundant contigs in the clustered contig set. The longest contig set consisted of the longest contigs from each subcomponent. Subcomponents were created by Trinity, taking into account splice variants. Thus, they included one or more contigs. However, if two or more subcomponents were created from one gene, it was predicted that the longest contigs would be selected from the multiple subcomponents, and therefore, the contigs in the multiple subcomponents would remain as redundant contigs. In contrast, annotated contigs were created by selecting the contig exhibiting the highest homology to a gene from among the various contigs exhibiting homology to that gene. Thus, it was predicted that partial contigs and incomplete contigs would be removed.

### Redundant and erroneous contigs lead to inaccurate detection of DECs and inaccurate GO slim term distribution for DECs

Our results suggested that the raw contig set contained redundant contigs. The redundant contigs were removed from the raw contig set to create the annotated contig set, but had not been removed adequately via approaches based on longest contigs or clustered contigs. In the annotated contig set, the correlation coefficient was increased compared with raw contig set (Fig. 1d), and erroneous contigs had been removed. There was not much difference in the number of DECs exhibiting co-identity with DEGs between the raw and annotated contig sets. However, the number of DECs that did not exhibit homology with DEGs in the annotated contig set was less than in raw contig set. Consistency in the GO distribution between DEGs and DECs was higher for the annotated contig set than the raw contig set. In contrast, the number of DECs not exhibiting co-identity with DEGs and the number of erroneous contigs were not decreased in the longest or clustered contig sets, which each still included redundant contigs. Additionally, consistency in GO distribution between DEGs and DECs was not improved in either of these reference sequences. These results suggested that the redundant contigs and erroneous contigs in the raw contig set were inaccurately detected as DECs, and were not identical to DEGs. These inaccurate DECs would cause inaccurate GO distribution of DECs.

### Removal of redundant contigs using an integrated plant protein database with application for unsequenced plant genomes

In this study, we selected *A. thaliana* as the model because its genome sequence has been determined and is accompanied by detailed gene information. The presence of redundant contigs was confirmed in primary contigs constructed by assembly of RNA-Seq reads of *A. thaliana*. Consequently, it was also revealed that the low detection accuracy of DECs was caused by redundant contigs. We proposed a method involving homology searches against the *A. thaliana* gene database for removing redundant and erroneous contigs from the contig set constructed by *de novo* assembly. Additionally, we confirmed that the low detection accuracy of DECs was eliminated when using the subset of contigs (annotated contigs) obtained by applying this method. However, when applying this method to a plant lacking a well-developed gene database, a protein or gene database of plants excluding the plant in question was required for homology searching. Removing redundancy from contigs of *A. thaliana* was tested using non-duplicative or duplicative combined protein database (PlantClust50 DB or Plant DB, respectively), which consisted of protein sequences of 14 species of plants. Using PlantClust50 DB, a reduction in contig number and an increase in average contig length and N50 value compared with the raw contig set were confirmed. However, these improvements were not observed when using PlantDB contig set. The number of multiple-hit contigs also was lower with the PlantClust50 DB contig set, but not with the Plant DB contig set (Table 2). This suggested that the set of Plant DB contigs had redundant contigs and that they could be removed by eliminating the redundant sequences in Plant DB. Compared with the log2-fold change plot of raw contigs vs. the gene dataset, the correlation coefficient was improved and the number of erroneous contigs was decreased by removing redundant contigs using searches against Plant DB and PlantClust50 DB (Table 4). These results suggested that the number of erroneous contigs was reduced in both the Plant DB and PlantClust50 contig sets.

### Accurate GO distribution of DECs detected after removing redundant contigs using non-duplicative plant protein database

When detecting DECs using either the Plant DB or PlantClust50 contig sets as reference sequences, the number of DECs exhibiting co-identity with DEGs was decreased. However, the number of DECs not identical with DEGs was also decreased (Fig. 2). We confirmed that the conformity of GO distribution of DECs to the GO distribution of DEGs was improved to the same extent as for the annotated contig set only with the PlantClust50 contig set (Figs. 3 and 4). The increase in the number of DECs not identical with DEGs was common to both the Plant DB and PlantClust50 contig sets. The cause of the increase was inferred to be the use of a protein database of plants excluding *A. thaliana* to identify contig redundancy. The protein database was estimated to contain well-conserved sequences, but presumably not to contain sequences unique to *A. thaliana*. If contigs to be detected as DECs possessed a sequence unique to *A. thaliana*, such a sequence could not exhibit homology with the protein sequences in the database. Thus, it was estimated that only contigs encoding the same functions as the contigs to be detected would be selected as DECs. Therefore, we assumed that the DECs identified as false positive in PlantClust DB contig set corresponded to the DEGs that could not be covered by the protein database.

When DECs were detected using PlantClust50 contig sets as reference sequences, the number of DECs exhibiting co-identity with DEGs was decreased from PlantClustDB. The GO distribution in DECs detected by PlantClust50 DB contigs fitted more closely to the distribution in DEGs than the distribution in DECs detected by Plant DB contigs. Therefore, it was suggested that the cause of decrease of identical DECs in PlantDB50 is that the contigs identical with DEGs were removed and the contigs which are resemble to the contigs identical with DEGs were selected from the clusters as representative sequences.

Additionally, we had set identity to 0.8 and 0.5 to form the non-duplicative database based on Plant DB. 38,741 contigs were generated with an identity setting of 0.8, and this number was closer to the number of transcript (35,385) than for either other setting. However, the 0.8 setting did not increase the number of DECs assigned to the GO terms in the contigs; 28 GO slim terms were significantly different in annotation count between DECs and DEGs in PlantClust DB. Significantly different GO terms in annotation count between DECs and DEGs were 30 and 17 in raw and PlantClust50 contig sets, respectively. This discrepancy was assumed to be result from the duplicate genes in the gene dataset. A more detailed study should reveal the appropriate identity setting.

## Conclusion

We designed and tested a method to create redundancy-removed contig sets suitable for comparative analysis with unsequenced plant genomes. Raw contigs were created by *de novo* assembly using *A. thaliana* RNA-Seq reads to confirm the accuracy of the assembled contigs. Redundant contigs in raw contig set were estimated and removed by our method, which involved BLAST searches; the resulting contig set was compared to sets created with each of two existing methods. The homology-based method identified redundancy in the raw contig set expressly and removed redundant contigs effectively. On the other hand, redundant contigs were not removed adequately by either of the two existing methods. Applying the homology-based method improved the detection accuracy of DEGs and distribution of GO terms in comparative RNA-Seq analysis significantly, demonstrating that the method can improve the possibility of detecting a useful gene by comparative analysis of RNA-Seq data in unsequenced plants.

## Abbreviations

agriGO: a GO analysis toolkit for the agricultural community
DEGs: Differentially expressed genes
TAIR: The Arabidopsis Information Resource

## References

[1] Cong L, Ran FA, Cox D, Lin S, Barretto R, Habib N (2013). Multiplex genome engineering using CRISPR/Cas systems. Science.

[2] Jacobsen E, Schouten HJ (2007). Cisgenesis strongly improves introgression breeding and induced translocation breeding of plants. Trends Biotechnol.

[3] Ye X, Al-Babili S, Klöti A, Zhang J, Lucca P, Beyer P (2000). Engineering the provitamin A (beta-carotene) biosynthetic pathway into (carotenoid-free) rice endosperm. Science.

[4] Fu SF, Chen PY, Nguyen QT, Huang LY, Zeng GR, Huang TL (2014). Transcriptome profiling of genes and pathways associated with arsenic toxicity and tolerance in Arabidopsis. BMC Plant Biol.

[5] Carbonell-Bejerano P, Rodríguez V, Royo C, Hernáiz S, Moro-González LC, Torres-Viñals M (2014). Circadian oscillatory transcriptional programs in grapevine ripening fruits. BMC Plant Biol.

[6] Canales J, Moyano TC, Villarroel E, Gutiérrez RA (2014). Systems analysis of transcriptome data provides new hypotheses about Arabidopsis root response to nitrate treatments. Front Plant Sci.

[7] Zhai R, Feng Y, Wang H, Zhan X, Shen X, Wu W (2013). Transcriptome analysis of rice root heterosis by RNA-Seq. BMC Genomics.

[8] Xu J, Yuan Y, Xu Y, Zhang G, Guo X, Wu F (2014). Identification of candidate genes for drought tolerance by whole-genome resequencing in maize. BMC Plant Biol.

[9] Schaffer RJ, Ireland HS, Ross JJ, Ling TJ, David KM (2013). SEPALLATA1/2-suppressed mature apples have low ethylene, high auxin and reduced transcription of ripening-related genes. AoB Plants.

[10] Wang Z, Gerstein M, Snyder M (2009). RNA-Seq: a revolutionary tool for transcriptomics. Nat Rev Genet.

[11] Kim KM, Park JH, Bhattacharya D, Yoon HS (2014). Applications of next-generation sequencing to unravelling the evolutionary history of algae. Int J Syst Evol Microbiol.

[12] Paux E, Sourdille P, Salse J, Saintenac C, Choulet F, Leroy P (2008). A physical Map of the 1-gigabase based Wheat chromosome 3B. Science.

[13] Zhang XM, Zhao L, Larson-Rabin Z, Li DZ, Guo ZH (2012). De novo sequencing and characterization of the floral transcriptome of dendrocalamus latiflorus (Poaceae: Bambusoideae). PLoS One.

[14] He M, Wang Y, Hua W, Zhang Y, Wang Z (2012). De novo sequencing of hypericum perforatum transcriptome to identify potential genes involved in the biosynthesis of active metabolites. PLoS One.

[15] Yang Y, Xu M, Luo Q, Wang J, Li H (2014). De novo transcriptome analysis of Liriodendron chinense petals and leaves by Illumina sequencing. Gene.

[16] Zerbino DR, Birney E (2008). Velvet: algorithms for de novo short read assembly using de Bruijn graphs. Genome Res.

[17] Schulz MH, Zerbino DR, Vingron M, Birney E (2012). Oases: robust de novo RNA-seq assembly across the dynamic range of expression levels. Bioinformatics.

[18] Zhao QY, Wang Y, Kong YM, Luo D, Li X, Hao P (2011). Optimizing de novo transcriptome assembly from short-read RNA-Seq data: a comparative study. BMC Bioinformatics.

[19] Li R, Zhu H, Ruan J, Qian W, Fang X, Shi Z (2010). De novo assembly of human genomes with massively parallel short read sequencing. Genome Res.

[20] Strickler SR, Bombarely A, Mueller LA (2012). Designing a transcriptome next-generation sequencing project for a nonmodel plant species. Am J Bot.

[21] Surget-Groba Y, Montoya-Burgos JI (2010). Optimization of de novo transcriptome assembly from next-generation sequencing data. Genome Res.

[22] Duan J, Xia C, Zhao G, Jia J, Kong X (2012). Optimizing de novo common wheat transcriptome assembly using short-read RNA-Seq data. BMC Genomics.

[23] Li W, Godzik A (2006). Cd-hit: a fast program for clustering and comparing large sets of protein or nucleotide sequences. Bioinformatics.

[24] Davidson NM, Oshlack A (2014). Corset: enabling differential gene expression analysis for de novo assembled transcriptomes. Genome Biol.

[25] O’Rourke JA, Yang SS, Miller SS, Bucciarelli B, Liu J, Rydeen A (2013). An RNA-Seq transcriptome analysis of orthophosphate-deficient white lupin reveals novel insights into phosphorus acclimation in plants. Plant Physiol.

[26] Altschul SF, Gish W, Miller W, Myers EW, Lipman DJ (1990). Basic local alignment search tool. J Mol Biol.

[27] Krasileva KV, Buffalo V, Bailey P, Pearce S, Ayling S, Tabbita F (2013). Separating homeologs by phasing in the tetraploid wheat transcriptome. Genome Biol.

[28] Lamesch P, Berardini TZ, Li D, Swarbreck D, Wilks C, Sasidharan R (2012). The Arabidopsis Information Resource (TAIR): improved gene annotation and new tools. Nucleic Acids Res.

[29] Du Z, Zhou X, Ling Y, Zhang Z, Su Z (2010). agriGO: a GO analysis toolkit for the agricultural community. Nucleic Acids Res.

[30] Langmead B, Salzberg SL (2012). Fast gapped-read alignment with Bowtie 2. Nat Methods.

[31] Mortazavi A, Williams BA, McCue K, Schaeffer L, Wold B (2008). Mapping and quantifying mammalian transcriptomes by RNA-Seq. Nat Methods.

[32] Conesa A, Götz S, García-Gómez JM, Terol J, Talón M, Robles M (2005). Blast2GO: a universal tool for annotation, visualization and analysis in functional genomics research. Bioinformatics.

[33] Dohm JC, Lottaz C, Borodina T, Himmelbauer H (2008). Substantial biases in ultra-short read data sets from high-throughput DNA sequencing. Nucleic Acids Res.

[34] Miller JR, Koren S, Sutton G (2010). Assembly algorithms for next-generation sequencing data. Genomics.

